# Epigenetic Regulation of Antifungal Drug Resistance

**DOI:** 10.3390/jof8080875

**Published:** 2022-08-19

**Authors:** Sandip Patra, Mayur Raney, Aditi Pareek, Rupinder Kaur

**Affiliations:** 1 Laboratory of Fungal Pathogenesis, Centre for DNA Fingerprinting and Diagnostics, Hyderabad 500039, India; 2 Graduate Studies, Regional Centre for Biotechnology, Faridabad 121001, India

**Keywords:** histones, histone methylation and acetylation, chromatin, multidrug transporter, azoles, echinocandins and polyenes, DNA mismatch repair, *FKS* gene mutations

## Abstract

In medical mycology, epigenetic mechanisms are emerging as key regulators of multiple aspects of fungal biology ranging from development, phenotypic and morphological plasticity to antifungal drug resistance. Emerging resistance to the limited therapeutic options for the treatment of invasive fungal infections is a growing concern. Human fungal pathogens develop drug resistance via multiple mechanisms, with recent studies highlighting the role of epigenetic changes involving the acetylation and methylation of histones, remodeling of chromatin and heterochromatin-based gene silencing, in the acquisition of antifungal resistance. A comprehensive understanding of how pathogens acquire drug resistance will aid the development of new antifungal therapies as well as increase the efficacy of current antifungals by blocking common drug-resistance mechanisms. In this article, we describe the epigenetic mechanisms that affect resistance towards widely used systemic antifungal drugs: azoles, echinocandins and polyenes. Additionally, we review the literature on the possible links between DNA mismatch repair, gene silencing and drug-resistance mechanisms.

## 1. Introduction

Invasive fungal infections are an increasing threat to human health worldwide, with approximately 150 million people afflicted with serious mycoses [1,2]. The mortality rate associated with fungal infections exceeds that of many diseases, including malaria [1,2,3,4]. A wide range of factors, including an ever-increasing high-risk population (patients with immune suppression, immune dysfunction, diabetes mellitus and patients carrying indwelling catheters or undergoing surgery and organ transplantation), indiscriminate usage of antibiotics, a limited antifungal arsenal and emerging resistance to current antifungal drugs, contribute to the increasing incidence of fungal infections [1,5,6]. Species belonging to the *Candida* genus are the most prevalent agents of hospital-acquired invasive fungal infections, while *Aspergillus*, *Cryptococcus* and *Penumocystis* spp. also contribute to life-threatening fungal infections in healthcare settings worldwide [1,2,4,7,8].

The spectrum of systemic antifungal drugs used to treat fungal infections currently encompasses three major classes, azoles, polyenes and echinocandins, which target either the fungal plasma membrane or cell wall [9]. Lanosterol 14-α demethylase, encoded by the *ERG11* gene in the *Candida* species and *Cyp51* genes in filamentous fungi, is the target of azole drugs, while β (1,3) glucan synthase, encoded by *FKS* genes, is the target of echinocandin antifungals (Table 1) [9]. Polyene drugs target the major sterol in the fungal plasma membrane, ergosterol (Table 1) [9].

The fungistatic action of azoles is primarily attributed to diminished ergosterol levels in the plasma membrane and intracellular accumulation of toxic sterol intermediates [9]. Ergosterol biosynthesis is a multi-enzyme pathway, with the Erg11 enzyme catalyzing the conversion of lanosterol to 4-4-Dimethylcholesta-8,14,24-trienol, and with sterol 14alpha-demethylases acting on lanosterol and/or eburicol in the pathogenic fungi of humans [9,10]. During azole exposure, toxic 14alpha-methyl metabolites are produced by the ergosterol biosynthesis pathway, due to the action of the Erg3 (C-5 sterol desaturase) enzyme [9,10,11]. Perturbed plasma-membrane permeability and integrity and the reduced activity of plasma-membrane proteins are also key manifestations of azole drugs [9,10,11,12]. In contrast to azoles, polyenes and echinocandins are usually fungicidal, with their toxicity being associated with impaired cell-wall biosynthesis and perturbed plasma-membrane function, respectively [9,11,12]. 

The above-mentioned limited therapeutic options are restricted further due to the development of resistance towards current antifungals in susceptible fungal isolates, and/or the emergence of drug-resistant fungal species, thereby rendering antifungal therapeutics ineffective [5,12,13]. The growing clinical challenge of antifungal therapy failure can stem from an interplay among host immune responses, antifungal drug characteristics, drug–drug interactions, different phenotypic or morphological forms of pathogens (including sessile and planktonic forms) and innate or acquired resistance towards administered drugs [5,11,12,13]. Recent studies on medically important fungi have recognized epigenetics as a key feature regulating biological processes involved in the adaptation to various stress conditions, switching between two colony forms, morphological plasticity involving yeast and filamentous forms, antifungal resistance and virulence [3,14,15,16,17,18]. In this review, we discuss the epigenetic mechanisms that aid in the development of resistance towards antifungal drugs commonly used in clinical settings.

## 2. Antifungal Resistance Mechanisms

In clinical settings, azole resistance is generally found at a much higher frequency than echinocandin resistance, while resistance to polyenes is seldom observed [5,13]. *Candida albicans* is the most common causative agent of bloodstream *Candida* infections, while *C. auris* and *C. glabrata* appear as drug-resistant *Candida* species, with *C. auris* showing resistance to all three antifungal classes and *C. glabrata* displaying resistance to azoles and echinocandins [4,7,19,20,21]. Notably, azole and echinocandin resistance has been reported for about 8% of *C. glabrata* clinical isolates [4], while 41% and 4% of *C. auris* isolates were found to be resistant to two and three antifungal classes, respectively [19].

**Figure 1:**
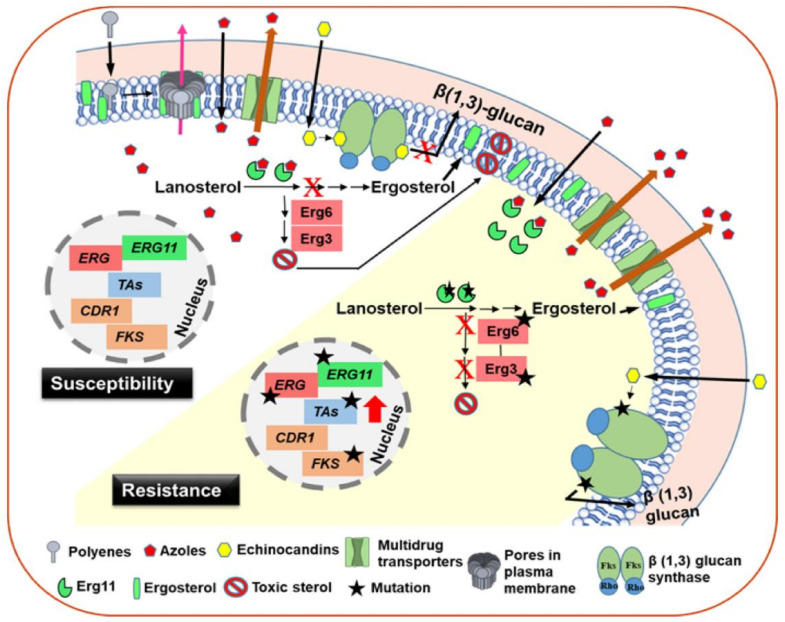
Prevalent resistance mechanisms towards azole, echinocandin and polyene antifungals. Gain-of-function mutations in the transcriptional activator (TA)-encoding genes (*TAC1*, *MRR1*, *PDR1* and *UPC2A*) lead to amplification of their respective target genes. Overexpression of multidrug transporter and *ERG11/Cyp51* genes are frequently observed in azole-resistant fungal isolates, as indicated by the red arrow.

The mechanisms that underlie antifungal drug resistance in medically important fungi are well-studied. These primarily include the transcriptional activation of multidrug efflux pumps, alteration in the drug target, overexpression of the drug target, changes in the cellular biosynthetic or stress response pathways and biofilm formation [10,11,12,13]. At the molecular level, these mechanisms include gain-of-function mutations in the transcriptional regulators of multidrug transporter genes (for azole drugs), overexpression or mutations in the azole-target enzyme-encoding *ERG11* or *Cyp51* genes (for azole drugs), mutations in the hot-spot regions of echinocandin target-encoding *FKS1-3* genes (for echinocandin drugs) and mutations in the ergosterol biosynthesis pathway (for polyenes) (Figure 1) [10,11,12,13]. In addition, aneuploidy, loss of heterozygosity, mutations in the DNA mismatch repair pathway, amplification of specific chromosome regions, formation of new chromosomes and multidrug transporter-associated but transporter-gene overexpression-independent hyper resistance, have also been associated with resistance to antifungal drugs in clinical settings [10,11,12,13,22,23].

Multiple mechanisms account for azole resistance in human fungal pathogens, with the overexpression of multidrug efflux pumps and membrane-associated transporters belonging to ATP-binding cassette transporter (ABC-T) and major facilitator transporter (MFS-T) superfamily, respectively, occupying the central stage [10,11,13]. These transporters actively pump azoles out, thereby substantially lowering the drug concentration inside the cell [10,11,12,13]. Gene amplification and/or gain-of-function mutations in their transcriptional activator (Zn-cluster proteins)-encoding genes (*TAC1* and *MRR1* (in *C. albicans*) and *PDR1* (in *C. glabrata* and *C. auris*)) contribute to the overexpression of azole transporters (Figure 1) [10,11,13]. This mechanism belongs to pleiotropic drug resistance (PDR) or multidrug resistance (MDR) regulatory network [10,11]. The common components of this drug-resistance system in *Candida* species are depicted in Table 2. 

Another prevalent azole-resistance mechanism involves overexpression or mutations in the azole target enzyme, Erg11/CYP51, with mutations decreasing enzyme-drug binding [10,11,13]. Gene amplification and gain-of-function mutations in the Zn2-Cys6 transcription factor Upc2 have been reported to result in the elevated expression of *ERG11*, with *C. albicans* and *C. glabrata* containing one (Upc2) and two(Upc2a and Upc2b) homologs of this regulator, respectively [10,11,13]. Additionally, mutations in the *ERG3* gene, whose product converts episterol to ergosta-5,7,24 (28)-trienol during ergosterol biosynthesis, have also been associated with azole resistance, as the Erg3 enzyme is required for the formation of toxic sterols during azole treatment [10,11].

The predominant mechanism for resistance towards echinocandins, which causes the non-competitive inhibition of the β(1,3)-glucan synthase enzyme, involves mutations in the highly conserved hot-spot regions of *FKS* genes (Figure 1) [11,13]. β(1,3)-glucan synthase consists of a transmembrane catalytic Fks subunit and intracellular regulatory Rho1 subunit, and synthesizes β(1,3)-glucan (structural cell-wall polysaccharide component) from uridine diphosphate glucose [9,11,24]. The impeded synthesis of β(1,3)-glucan results in defective cell-wall assembly and is fungicidal [9,11]. *FKS* gene mutations reduce the affinity of the Fks enzyme towards echinocandins [11,12,13].

Polyene drugs bind to ergosterol and may generate membrane-spanning pores, as well as extract ergosterol from the plasma membrane by forming large extramembranous aggregates (Figure 1) [11,13]. The fungicidal nature of polyenes is attributed to the loss of plasma-membrane potential, leakage of intracellular ions and oxidative damage [10,13]. Polyene resistance is rare in clinical settings and has been associated with the loss of or mutations in *ERG* genes, including *ERG2*, *ERG3*, *ERG6* and *ERG11* in *Candida* species [5,10,13]. These perturbations in the ergosterol biosynthesis pathway either reduce the ergosterol amount or replace ergosterol with alternative precursor sterols in the cell membrane [10,13]. Amphotericin B resistance in *C. tropicalis* and *Aspergillus terreus* was found to be associated with higher levels of β(1,3)-glucan and increased activity of the reactive oxygen species (ROS)-detoxifying enzyme catalase, respectively [25,26]. Compared to azoles and echinocandins, polyenes can produce severe side-effects, largely due to their affinity towards mammalian cholesterol [9,10,13].

Since the genetic and molecular basis of antifungal drug-resistance mechanisms is well-studied and reviewed in detail in recent articles [10,11,13,22,23], this review focuses on the epigenetic regulation of prevalent antifungal resistance mechanisms, with a special focus on *Candida* spp.

## 3. RNA-Based Epigenetic Modifications in Antifungal Resistance

Epigenetic modifications refer to the heritable and stable cellular changes that are not caused due to alterations in the DNA sequence [27]. These modifications, which are primarily mediated by DNA, RNA or chromatin, transiently modulate gene activity, without changing DNA or protein sequences [27]. RNA-based epigenetic mechanisms broadly consist of RNAi (RNA interference)- and lncRNAs (long non-coding RNAs)-based systems [27,28]. LncRNAs are RNA molecules that are more than 200 nucleotides long, and are predominantly transcribed by RNA polymerase II [29]. Many lncRNAs undergo mRNA-like processing, including polyadenylation and splicing, and are degraded by exosomes [29]. 

The RNAi pathway is governed by small RNAs (sRNAs) that are generated by RNA-dependent RNA polymerases and processed by the Dicer endonuclease [28]. The incorporation of the processed sRNAs into the Argonaute complex leads to the targeting of selective complementary mRNAs, resulting in the translation inhibition or degradation of the target mRNA [28]. In *Mucor circinelloides* (a cause of mucormycosis), the RNAi-based epigenetic drug-resistance mechanism, epimutation, has been shown to confer resistance to FKBP12 (peptidyl-prolyl isomerase)-binding antifungal agents, FK506 and rapamycin, which block calcineurin and TOR signaling pathways, respectively [30], the *fkbA* gene codes for the FKBP12 protein. The degradation of *fkbA* mRNA, due to the core RNAi machinery-dependent endogenous expression of sRNAs against the *fkbA* gene, was found to be the mechanism underlying FK506- and rapamycin-resistant strains of *M. circinelloides* [30]. This mechanism was reported to be both reversible and epigenetic in nature [30]. Similarly, sRNA production against the *pyrF/pyrG* (*URA5/URA3*) gene in *M. circinelloides* accounted for resistance against the antifungal compound 5-fluoroorotic acid (5-FOA), as products of *pyrF/pyrG* genes are involved in the conversion of FOA into a toxic compound [31]. These reports suggest that the epimutant appearance may reflect a prevalent mechanism that allows the survival of various stresses, including antifungal drugs, in *M. circinelloides* [30,31]. Furthermore, although the RNAi system has also been implicated in the recruitment of heterochromatin proteins to target genes, which culminates in impeded gene expression [28,32], the significance of this function of the RNAi machinery in antifungal resistance is yet to be determined. Additionally, the role of RNAi in antifungal resistance in other human pathogenic fungi remains elusive.

LncRNAs have been reported to modulate drug resistance in the fission yeast *Schizosaccharomyces pombe* through transcriptional interference [33]. It has been reported that *TGP1* (codes for a glycerophosphodiester transporter 1) gene expression is controlled by the lncRNA, *ncRNA.1343*, via increased nucleosome density at the *TGP1* locus, with *ncRNA.1343* deletion leading to susceptibility to many drugs, including hydroxyurea and caffeine [33]. However, the role of lncRNAs in resistance towards azole, echinocandin and polyene antifungals in medically important fungi is yet to be investigated. 

## 4. Chromatin-Based Epigenetic Modifications in Antifungal Resistance

A eukaryotic cell compacts its genetic material inside the nucleus as nucleosomes, the basic unit of eukaryotic chromatin, by wrapping 146 bp of negatively charged DNA around a positively charged octameric protein complex of four types of histone proteins: H2A, H2B, H3 and H4 [34,35]. A canonical nucleosome contains two copies of histone H2A, H2B, H3 and H4, in which histones H2A and H2B create a dimer of dimer that interacts with a tetramer of two molecules: histones H3 and H4 [34,35]. The linker histone protein H1 binds to the linker DNA between two nucleosomes, thereby linking two consecutive nucleosomes [34,35]. Genome-wide nucleosome positioning and chromatin modifications play an important role in DNA-related cellular processes, including transcription and recombination [35,36].

Chromatin modifications that involve chemical or structural changes in the chromatin are represented by modifications of DNA (the methylation of cytosine and adenine bases), post-translational modifications of histones (arginine and lysine methylation, lysine SUMOylation, ubiquitination, acetylation, ribosylation and biotinylation, arginine citrullination, proline isomerization and serine/threonine/tyrosine phosphorylation) and conformational changes owing to the reconfiguration of chromatin by ATP-dependent chromatin-remodeling complexes [35,36]. These epigenetic modifications regulate gene expression by controlling the accessibility to chromatin by components of the transcriptional machinery [35,36].

### 4.1. Histone-Modifying Enzymes in Antifungal Resistance

The two major histone post-translational modifications, the methylation and acetylation of lysine residue in the N-terminal tail of histone proteins, were assessed for their roles in antifungal resistance and virulence in human fungal pathogens [14,37]. Both lysine acetylation and methylation are dynamic reversible modifications, with acetylated and methylated histones frequently representing open and closed chromatin states, via the de-condensation and compaction of the chromatin structure, respectively [35,36]. These modifications impact gene expression by governing the access of transcriptional regulatory proteins, including transcriptional factors and chromatin remodelers, to the chromosomal DNA [36,38]. Specifically, acetylation neutralizes the positive charge of lysines in histone proteins, which results in reduced electrostatic attraction between histones and negatively charged DNA [38]. This facilitates the unwinding of DNA, thereby providing access to the cellular machinery governing DNA-related processes [35,38]. In contrast, methylation, which involves the addition of one, two or three methyl groups in the same lysine, does not alter the charge in histone proteins [38]. Instead, histone methylation modifies the nucleosomes’ properties and acts as an activating (enables the looser winding of DNA around histones) or repressive (enables the tighter wrapping of DNA around histones) mark by facilitating or limiting the access of the cellular machinery to DNA, respectively [36,38].

The lysine acetyltransferases (KATs) and histone acetyltransferases (HATs) perform the acetylation of the lysine residue in cellular and histone proteins, respectively, while lysine methylation in histone proteins is mediated by histone methyltransferases (HMTs) [14,36,37,38]. Similarly, demethylation and deacetylation reactions that involve the removal of acetyl and methyl groups from cellular proteins are mediated by lysine demethylases (KDMs) and lysine deacetylases (KDACs) [14,36,37,38]. The histone-modifying KDACs are referred to as HDACs [14,36,37,38]. The known functions of the fungal enzymes that regulate the dynamic modifications of acetylation and methylation in histone proteins involved in antifungal resistance are described below.

#### 4.1.1. Histone Acetyltransferases

Histone acetylation has been implicated in antifungal resistance in *C. albicans* and *C. glabrata* [14,17,39,40,41,42]. The deletion of the gene coding for histone acetyltransferase 1 (Hat1), which acetylates histone H4 at lysines 5 and 12 prior to its incorporation into the chromatin, led to caspofungin sensitivity in *C. albicans*, which was attributed to elevated ROS production due to caspofungin [40]. The deletion of *HAT1* or *HAT2*, which code for regulatory subunits of the chromatin assembly associated acetyltransferase complex NuB4, led to voriconazole and itraconazole resistance [41], suggesting opposite roles for Hat1 in regulating azole and echinocandin resistance. 

Interestingly, a reduced histone H4 dosage has also been associated with enhanced caspofungin sensitivity, which was rescued by the exogenous addition of vitamin C, probably due to its antioxidant properties [40]. Two redundant FK506-binding proteins, CgFpr3 and CgFpr4, which maintain histone H3 and H4 levels, were recently shown to negatively regulate *CgPDR1*-network genes and azole resistance in *C. glabrata* [43]. Moreover, fluconazole exposure was found to increase the levels of histones H3 and H4 in the same study [43]. Collectively, these findings underscore the contribution of histone abundance and deposition to the modulation of antifungal resistance, although the underlying mechanism is yet to be deciphered.

Furthermore, the histone H3K56 acetyl transferase Rtt109, which acetylates histone H3 at the lysine 56 residue, was implicated in antifungal drug resistance, as *RTT109* deletion in *C. albicans* led to an elevated susceptibility towards two echinocandins, caspofungin and micafungin, and nucleic acid synthesis-inhibitory antifungal 5-fluorocytosine [40,44,45]. *RTT109* deletion in *C. glabrata* was also reported to result in increased caspofungin sensitivity, as identified in a Tn*7* transposon-insertion mutant library screen for altered caspofungin susceptibility [46]. However, unlike *C. albicans*, *RTT109* deletion in *C. glabrata* was found to be associated with increased sensitivity towards fluconazole [39,44]. Recently, the histone acetyltransferase inhibitor CPTH2 (Cyclopentylidene-[4-(4-chlorophenyl)thiazol-2-yl)hydrazone) was shown to be fungicidal for *C. albicans* and have selective growth-inhibitory activity against the *Candida* species belonging to the CTG clade [47].

The fungal lysyl acetyltransferase, Gcn5, a component of many multi-subunit regulatory complexes, including SAGA and SLIK complexes, was recently implicated in caspofungin resistance, but not azole resistance in *C. albicans* [48]. Moreover, the increased susceptibility of the *gcn5Δ/Δ* mutant towards caspofungin was not due to high intracellular ROS levels [48]. In this context, it is noteworthy that the loss of another subunit of the SAGA/ADA coactivator complex, Ada2, resulted in increased fluconazole sensitivity in *C. albicans*, with an *ada2Δ/Δ* mutant displaying reduced levels of H3K9 acetylation at the *MDR1* locus, and impaired activation of fluconazole-induced *MDR1* gene expression [49]. Similarly, although the deletion of the *CgADA2* gene, which catalyzes H3K9 acetylation in *C. glabrata*, resulted in sensitivity to all three drugs, azoles, polyenes and echinocandins, the expression of CgPdr1-dependent multidrug-resistance genes was not perturbed in the *Cgada2Δ* mutant [50]. 

Furthermore, Gcn5 in *C. glabrata* was also recently implicated in the development of drug resistance. *CgGCN5* deletion led to both fluconazole and micafungin sensitivity, and mitigated against the emergence of drug resistance towards these drugs [51,52]. Of note, *CgGCN5* deletion was also found to be lethal with gain-of-function *CgPDR1* alleles that conferred fluconazole resistance to *C. glabrata* cells [51], underscoring the regulatory link between CgPdr1-mediated multidrug resistance and CgGcn5 complex-mediated chromatin remodeling. In summary, these findings point towards a complex system of histone acetylation-based regulation of drug-resistance genes. Further studies are needed to understand the mechanistic basis underlying the disparate drug susceptibility phenotypes associated with the alterations in components of histone acetylation and deacetylation machinery.

#### 4.1.2. Histone Deacetylases

**Figure 2:**
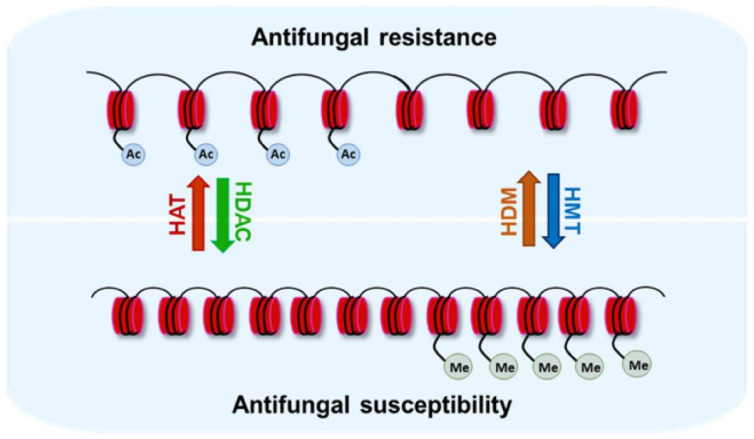
The effects of histone acetylation and methylation on resistance to antifungal drugs. Open and compact chromatin has been associated with decreased and increased drug susceptibility, respectively, in some cases.

In addition to HATs, HDACs have also been shown to govern antifungal resistance [14]. *C. albicans* azole-resistant isolates were found to have an increased expression of histone deacetylase-encoding genes, *HDA1* and *RPD3* [42]. Elevated *HDA1* and *RPD3* expressions in fluconazole-resistant strains were reversed upon the development of stable azole resistance during the course of acquired fluconazole resistance in vitro [42], thereby underscoring a transient requirement of histone acetylation in the antifungal resistance process (Figure 2). Furthermore, Hda1 and Rpd3 were shown to control Hsp90-dependent azole resistance in *S. cerevisiae*, as *HDA1* and *RPD3* deletions led to the diminished functioning of the heat-shock protein, Hsp90, with the acetylation of Hsp90 controlling the activity of this highly conserved cellular chaperone [53]. In *C. albicans*, the simultaneous loss of four KDACs (Hos2, Hda1, Rpd3 and Rpd31) was required to abolish Hsp90-dependent fluconazole resistance [54]. Of note, Hsp90 has also been reported to facilitate the emergence of antifungal resistance through its effector molecule, calcineurin, which regulates many fungal stress-response pathways [55]. Consistent with this, the chemical inhibition of KDACs, Hsp90 or calcineurin blocks the emergence of antifungal resistance in many fungal pathogens [53,54,55]. Furthermore, the acetylation of the lysine 27 residue in Hsp90 has been shown to be critical for voriconazole and caspofungin resistance in *A. fumigatus* [56].

In *C. glabrata*, the loss of the NAD^+^-dependent histone deacetylase Hst1 led to fluconazole resistance, which was abrogated upon the deletion of *CgPDR1* or *CgCDR1* (encodes a major multidrug efflux pump) genes in the *Cghst1Δ* mutant, indicating an essential requirement for *CgCDR1* or *CgPDR1* in the CgHst1-dependent cellular response to azoles [57]. Furthermore, a *Cghst1Δ* mutant was found to have elevated *CgCDR1* and *CgPDR1* transcript levels, suggesting that CgHst1 acts as a negative regulator of the *CgPDR1* network genes [57,58]. Consistently, the nicotinamide-mediated inhibition of CgHst1 led to the enhanced transcription of *CgCDR1* and *CgPDR1* genes, and rendered *C. glabrata* cells resistant to fluconazole [57]. Similarly, the deletion of the *CaHST3* gene that codes for a NAD^+^-dependent histone deacetylase, led to echinocandin resistance in *C. albicans* [45]. In addition, four core subunits (CaSet3, CaHos2, CaSnt1 and CaSif2) of the histone deacetylase Set3 complex were found to control resistance in sessile biofilm-forming *C. albicans* cells against caspofungin and amphotericin B drugs [59].

Importantly, and consistent with the genetic analysis, the HDAC inhibitor trichostatin A was found to increase the sensitivity of *C. albicans* cells to azoles, due to the highly diminished transcriptional activation of *ERG1*, *ERG11*, *CDR1* and *CDR2* genes in response to azole exposure [60]. Similarly, the Hos2 HDAC inhibitor MGCD290 and azole drugs (fluconazole, voriconazole and posaconazole) were shown to have a synergistic growth-inhibitory effect on azole-resistant isolates of several fungal species (*Candida*, *Fusarium*, *Aspergillus*, *Rhizopus* and *Mucor*) [61]. Collectively, these findings highlight the possibility that the disruption of the critical balance between the acetylation and deacetylation of histone proteins may have a significant impact on antifungal resistance. A systematic analysis is required to identify key lysine residues in specific histone proteins for the ultimate clinical application of this strategy.

#### 4.1.3. Histone Methyltransferases and Demethylases

In comparison to HATs and HDACs, the role of HMTs and KDMs in drug resistance in pathogenic fungi is just beginning to be studied. Two recent studies have shown that the deletion of histone H3K4 methyltransferase (CgSet1)- and H3K36 methyltransferase (CgSet2)-encoding genes rendered *C. glabrata* cells more and less susceptible to azoles, respectively [43,62], highlighting the antagonistic roles of lysine 4 and 36 methylations in histone H3 in regulating azole resistance. Furthermore, while CgSet1-dependent azole resistance was attributed to the azole-induced transcriptional activation of *ERG* genes, including the azole target-encoding gene *ERG11*, a slight increase in the expression of *PDR1*-network genes in the *Cgset2Δ* mutant was observed that may contribute to decreased fluconazole susceptibility of the mutant [43,62]. Consistent with this idea, the histone demethylase CgRph1 in *C. glabrata* was found to control the expression of *PDR1*-network genes, with the *Cgrph1Δ* mutant exhibiting a low basal expression of *CgPDR1* and *CgCDR1* genes, as well as an increased susceptibility towards fluconazole [43]. The common chromatin modifiers and their known associations with azole and echinocandin drugs in *C. albicans* and *C. glabrata* are summarized in Table 3.

Collectively, these studies suggest that the methylation of histone H3 at lysine 4 and 36 residues is important for the control of the most prevalent azole-resistance mechanism in the *Candida* species. However, the role of histone H3 methylation in drug resistance in other fungal pathogens remains to be examined. In this context, it is noteworthy that heterochromatin-mediated gene silencing in the yeast *S. pombe* has recently been shown to confer unstable resistance to caffeine, with caffeine-resistant strains also exhibiting fluconazole and clotrimazole resistance [63]. Additionally, the loss of the JmjC domain histone demethylase Epe1 (a negative regulator of H3K9 methylation-dependent heterochromatin) increased the frequency of fluconazole-resistant colonies in *S. pombe*, which was found to be dependent on the presence of the H3K9 methyltransferase Clr4 [63,64]. These studies suggest that perturbing histone methylation-dependent gene silencing may hold promise for a new antifungal therapy.

Although the field of histone modification-based control of antifungal drug resistance is still in its infancy, there is growing evidence that this cellular process is an important resistance determinant in fungal pathogens. 

### 4.2. Chromatin-Remodeling Complexes in Antifungal Resistance

ATP-dependent chromatin-remodeling complexes play a key role in the establishment and maintenance of precise global nucleosome positioning, which is pivotal to gene-regulatory processes, including transcription initiation and DNA repair [65]. Chromatin remodelers utilize a helicase-like motor to modify the chromatin structure, which involves the disruption of nucleosome-DNA contacts, displacement or exchange of nucleosomes or mobilization of nucleosomes along DNA [65]. Each chromatin-remodeling complex possesses an ATPase subunit that belongs to the SF2 family of DEAD/H-box helicases and may aid in ATP-dependent translocation along DNA [65,66]. Based on the ATPase subunit, a characteristic of ATP-dependent chromatin-remodeling complexes, these complexes are classified into four major subfamilies: SWI/SNF (switch/sucrose non-fermentable), CHD (chromodomain helicase DNA binding), ISWI (imitation switch) and INO80 (inositol auxotroph 80) [65,66]. In addition to the ATPase subunit, chromatin-remodeling complexes contain other subunits, with these accessory subunits being pivotal to the modulation of ATPase domain activity, targeting DNA or modified histones and aiding the binding of the complex to the transcription factors [65,66].

The SWI/SNF complex has been implicated in antifungal resistance, with ATP-dependent chromatin-remodeling complexes acting as co-activators of transcriptional factors governing the expression of multidrug-resistance genes [65,67]. CaSnf2, the ATPase subunit of the SWI/SNF complex, has been shown to maintain an open chromatin, via histone displacement and nucleosome depletion, to facilitate the occupancy of transcription factor Mrr1 at the promoter of the drug transporter-encoding *MDR1* gene in *C. albicans* [68]. Consistently, *CaSNF2* deletion abolished elevated *CaMDR1* expression and led to the attenuation of fluconazole resistance in mutants carrying gain-of-function mutations in the *CaMRR1* gene [68]. *CaSNF2* deletion was also found to be associated with a modest increase in fluconazole susceptibility [68].

Recently, CgSnf2 and CgRtt106 (histone chaperone) have been shown to bind to the Cg*CDR1* gene promoter and control the azole-induced expression of *PDR*-network genes in *C. glabrata*, with *Cgrtt106Δ* and *Cgsnf2Δ* mutants also displaying increased azole susceptibility [67]. Therefore, chromatin architecture, as well as supply and post-translational modifications of histones, play important roles in governing the expression of key multidrug-resistance genes in two prevalent *Candida* pathogens: *C. albicans* and *C. glabrata*.

## 5. Role of DNA-Damage Repair Mechanisms in Antifungal Resistance

The exposure to fungicidal antifungals could lead to DNA damage due to elevated ROS production [69], with DNA damage being associated with genomic instability, loss of heterozygosity, aneuploidy due to defective chromosome segregation and genomic rearrangements [70]. Therefore, DNA repair mechanisms are likely to play a role in regulating antifungal resistance. DNA double-strand breaks can be repaired through the error-prone non-homologous end-joining (NHEJ) mechanism, wherein broken ends are processed and re-joined. This may result in insertions/deletions. DNA double-strand breaks can also be repaired through the accurate homologous recombination (HR) pathway wherein the repair of cut ends is performed using a homologous sequence as a template [70,71]. The DNA mismatch repair (MMR) pathway is involved in correcting DNA mismatches that have arisen from misincorporation errors of the DNA polymerase during DNA replication and/or stemmed from HR between two diverged DNA sequences [71]. The mismatch repair proteins, Msh2-3 and Msh2-6, are involved in the recognition of DNA mismatches, followed by either a repair or anti-recombination event involving the unwinding of DNA [71].

Fluconazole exposure has been shown to result in a loss of genetic heterozygosity, aneuploidy, isochromosome formation in *C. albicans* and formation of disomies in multiple chromosomes in *C. neoformans*, with these outcomes being associated with the development of fluconazole resistance due to an increase in the copy number of key antifungal resistance-conferring genes, including *CDR1* and *ERG11* [70,72]. Therefore, the inhibition of cellular DNA repair mechanisms may potentially enhance the activity of current antifungal drugs. Consistent with this notion, the loss of proteins that are involved in the double-strand break repair, CaMre11 and CaRad50 (HR and NHEJ pathway proteins), CgKu80 (NHEJ pathway protein) and CaRad52 (HR pathway protein), led to elevated fluconazole susceptibility, while the lack of mismatch repair proteins, CaMsh2 and CaPms1, and CaRad50, increased the incidence of appearance of fluconazole-resistant colonies in *C. albicans* [73,74]. In this context, it is worth noting that the deposition of the histone H3–H4 dimer at DNA damage sites has been shown to be, in part, dependent upon the histone acetyltransferase Hat1 [41].

Furthermore, the emergence of resistance to all three classes of drugs, caspofungin, fluconazole and amphotericin B, occurred at an elevated rate in the *C. glabrata msh2∆* mutant, with the drug-resistant clinical isolates of *C. glabrata* containing nonsynonymous mutations in the *CgMSH2* gene, resulting in a hypermutable (increased mutation rate) phenotype [75]. However, a link between *CgMSH2* mutations and resistance to antifungal drugs in clinical isolates collected from India, France, Spain and Korea was not observed in subsequent studies [76,77,78,79], raising the possibility that the MMR pathway-dependent multidrug-resistance phenotype is probably specific to a set of strains/isolates and may not be a universal antifungal resistance mechanism in *C. glabrata*. The DNA repair proteins that are associated with antifungal drug resistance in *C. albicans* and *C. glabrata* are listed in Table 4.

**Figure 3:**
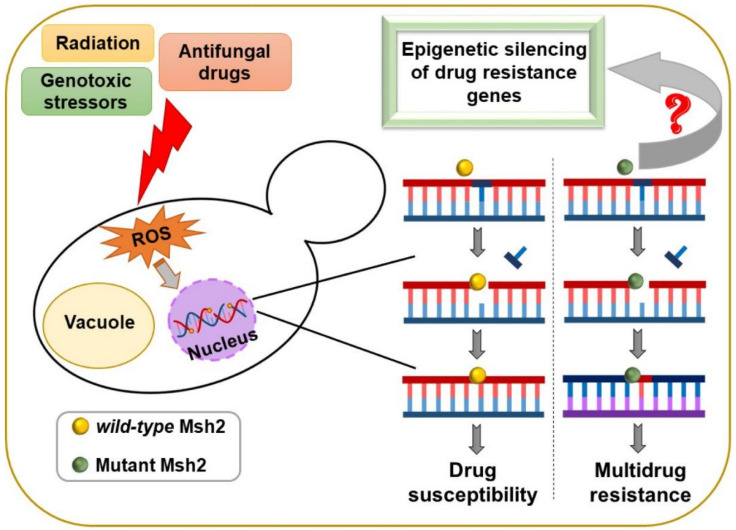
A possible link between the DNA mismatch repair system and antifungal resistance mechanisms.

Notably, MMR pathway proteins have also been associated with drug resistance and hypermutable phenotypes in other important fungal pathogens, *Cryptococcus* spp. and *A. fumigatus* [80,81,82,83,84]. Similarly, a mutation in the DNA polymerase delta subunit-encoding *POL3* gene led to a hypermutator phenotype in *Cryptococcus deneoformans*, with this mutation being mapped to the exonuclease proofreading domain [85]. These reports highlight that a dysfunction in DNA repair pathways may increase the mutation rate, which may enhance the cellular ability to rapidly acquire and maintain antifungal resistance-conferring mutations in healthcare settings (Figure 3). Furthermore, a recent study in *S. pombe* reported that MMR proteins, Pms1, Mlh1 and Msh2, are pivotal to epigenetic silencing at the mating-type HMR locus and telomeric silencing [86]. This link between MMR pathway perturbation and control of epigenetic silencing was attributed to the relocalization of Sir2 deacetylase from the silent loci to the rDNA regions, and the consequent increase in acetylation of histone H3 at lysines 14 and 56, and H4 at lysine 16, at HMR locus and telomeres [86]. Importantly, the loss of the silencing phenotype in *pms1Δ*, *mlh1Δ* and *msh2Δ* mutants was not due to increased mutation frequency [86]. These findings collectively raise the possibility that the elevated emergence of antifungal resistance upon the impairment of the MMR pathway in human fungal pathogens could arise from defects in the epigenetic regulation of drug-resistance gene expression (Figure 3). Further investigations are needed to test this hypothesis.

## 6. Conclusions

RNA forms, numbers of histone proteins, dynamic acetylation and methylation of histone proteins, acetylation of Hsp90 and probably the precise and timely functions of DNA repair proteins upon the recruitment of gene-silencing complexes aid in controlling resistance to cell-wall- and cell-membrane-targeting antifungal drugs in medically important fungi. This is consistent with epigenetic processes playing an essential role in adaptation to varied stress conditions in diverse organisms [14,18,87,88]. The transient drug-resistance-conferring epigenetic changes may give rise to and/or be followed by stable genomic alterations, including the amplification of multidrug-resistance and stress-responsive genes, which may enhance the virulence of the human pathogenic fungi. Therefore, a mechanistic understanding of epigenetic modifications will advance our understanding of fungal virulence mechanisms. Importantly, with the increased incidence of fungal infections and emergence of drug-resistant fungal pathogens, the expansion and strengthening of the current armory of antifungal drugs is urgently needed. In this regard, the epigenetic regulation of drug resistance is an avenue for further research. Finding agents that target fungal-specific histone-modifying enzymes and/or fungal-specific gene-silencing mechanisms could potentially provide new classes of antifungal drugs.

## Figures and Tables

**Table 1:** Major antifungal drugs and their cellular targets.

Antifungal Drug Classes	Target Pathway	Mode of Action	Commonly Used Drugs
Azoles	Ergosterol biosynthesis	Inhibit the activity of lanosterol 14α-demethylase, encoded by *ERG11* and *Cyp51* genes	Ketoconazole, fluconazole, voriconazole, itraconazole and posaconazole
Echinocandins	1,3 β-glucan biosynthesis	Inhibit the activity of β (1,3)-glucan synthase enzyme, encoded by *FKS* genes	Caspofungin, micafungin and anidulafungin
Polyenes	Ergosterol biosynthesis	Extract ergosterol and create pores in the plasma membrane	Amphotericin B, nystatin and natamycin

**Table 2:** Major multidrug transporters associated with azole resistance.

Organism	Efflux Pump	Pump Type
*Candida albicans*	CaCdr1	ABC-T
	CaCdr2	ABC-T
	CaMdr1	MFS-T
*Candida glabrata*	CgCdr1	ABC-T
	CgCdr2/CgPdh1	ABC-T
	CgSnq2	ABC-T
*Candida krusei*	CkAbc1	ABC-T
	CkAbc2	ABC-T
*Candida tropicalis*	CtCdr1	ABC-T
*Candida auris*	CauCdr1	ABC-T
	CauMdr1	MFS-T

**Table 3:** List of histone modifiers and their roles in antifungal drug resistance.

	Histone Modifier	Reported *Candida* Species Having Histone Modifier	Modification	Mutant Phenotype towards Antifungal Drugs		References
				Azoles	Echinocandins	
**Acetyl transferases**	Gcn5	*Ca*^#^, *Cg*^#^	H3K14 acetylation		Sensitivity in *Ca*	[17]
	Hat1	*Ca*, *Cg*		Resistance in *Ca*		[41]
	Rtt109	*Ca*, *Cg*	H3K56 acetylation		Sensitivity in *Ca*	[45]
	Ada2	*Ca*, *Cg*	SAGA complex subunit	Sensitivity in *Ca*		[17]
**Deacetylases**	Hos2 *	*Ca*, *Cg*		Sensitivity in *Ca*		[54]
	Rpd3 *	*Ca*, *Cg*		Sensitivity in *Ca*		[42,54]
	Rpd31 *	*Ca*		Sensitivity in *Ca*		[54]
	had1 *	*Ca*, *Cg*	H3K14 deacetylation	Sensitivity in *Ca*		[42,54]
	Hst1	*Ca*, *Cg*		Resistance in *Cg*		[57]
**Methyl transferases**	Set1	*Ca, Cg*	H3K4 methylation	Sensitivity in *Cg*		[62]
	Set2	*Ca, Cg*	H3K36 methylation	Resistance in *Cg*		[43]
**Demethylase**	Rph1	*Ca, Cg*		Sensitivity in *Cg*		[43]

* The redundant function in fluconazole resistance. ^#^ *Ca* and *Cg* refer to *Candida albicans* and *Candida glabrata*, respectively.

**Table 4:** DNA-damage repair genes implicated in antifungal drug resistance in *C. albicans* (*Ca*) and *C. glabrata* (*Cg*).

DNA Repair Pathway	Gene Name	Name Description	*Candida* Species Reported to Mutations	Mutant Phenotype towards Antifungals		
				Azoles	Echinocandins	Polyenes
Mismatch repair (MMR) pathway	*MSH2*	MutS homolog	*Ca, Cg*	Fluconazole resistance in *Ca* and *Cg* [73,75]	Caspofungin sensitivity in *Ca*, but caspofungin and micafungin resistance in *Cg* [73,75,76]	Amphotericin B resistance in *Cg* [75]
	*PMS1*	Post-meiotic segregation	*Ca*	Fluconazole resistance in *Ca* [73]	-	-
Homologous recombination (HR) pathway	*RAD52*	Radiation sensitive	*Ca*	Fluconazole susceptibility in *Ca* [73]	-	-
	*RAD50*	Radiation sensitive	*Ca, Cg*	Fluconazole susceptibility in *Ca*, but resistance in *Cg* [73,75]	Caspofungin sensitivity in *Ca*, but resistance in *Cg* [73,75]	Amphotericin B resistance in *Cg* [75]
	*MRE11*	Meiotic recombination	*Ca*	Fluconazole sensitivity in *Ca* [73]	-	-
Non-Homologous End-Joining (NHEJ) pathway	*YKU80*	Yeast Ku protein	*Ca, Cg*	No role in azole resistance in *Ca*, but increased fluconazole tolerance in *Cg* [73,74]	No role in caspofungin susceptibility in *Cg* [74]	-
